# Identification of Lactate-Related Gene Signature for Prediction of Progression and Immunotherapeutic Response in Skin Cutaneous Melanoma

**DOI:** 10.3389/fonc.2022.818868

**Published:** 2022-02-21

**Authors:** Yalin Xie, Jie Zhang, Mengna Li, Yu Zhang, Qian Li, Yue Zheng, Wei Lai

**Affiliations:** Department of Dermato-Venereology, The Third Affiliated Hospital of Sun Yat-sen University, Guangzhou, China

**Keywords:** skin cutaneous melanoma, prognostic signature, lactate, immunotherapy, TCGA, GEO

## Abstract

Skin cutaneous melanoma (SKCM) is a skin cancer type characterized by a high degree of immune cell infiltration. The potential function of lactate, a main metabolic product in the tumor microenvironment (TME) of SKCM, remains unclear. In this study, we systemically analyzed the predictive value of lactate-related genes (LRGs) for prognosis and response to immune checkpoint inhibitors (ICIs) in SKCM patients included from The Cancer Genome Atlas (TCGA) database. Cluster 3, by consensus clustering for 61 LRGs, manifested a worse clinical outcome, attributed to the overexpression of malignancy marks. In addition, we created a prognostic prediction model for high- and low-risk patients and verified its performance in a validation cohort, GSE65904. Between TME and the risk model, we found a negative relation of the immunocyte infiltration levels with patients’ risk scores. The low-risk cases had higher ICI expression and could benefit better from ICIs relative to the high-risk cases. Thus, the lactate-related prognosis risk signature may comprehensively provide a basis for future investigations on immunotherapeutic treatment for SKCM.

## Introduction

Skin cutaneous melanoma (SKCM) is more aggressive than other skin cancer types owing to its rapid progression, poor prognosis, and high mortality (1). Although the cases invasive melanoma account for ~5% of all skin malignant tumors, it causes >75% of skin cancer-related deaths. The five-year survival rates in localized or regional melanoma are 98% and 64%, respectively, however, these rates reduce to 23% in the advanced stages (2), thereby illustrating that early intervention to prevent the disease from metastasizing is essential for improving the clinical prognoses. In the early stages, surgery is the most effective curative strategy, while for the metastatic cases, systemic treatment plays a significant role in inhibiting further disease progression (3).

In recent years, immunotherapy has emerged as the most promising treatment modality against several tumor types. Immunotherapy comprises therapeutic strategies that target various components and signal pathways of the immune system (4). The specific mechanism of action is based on disrupting the tolerogenic nature of human cancer and rebooting the antitumor effects exerted on the tumor microenvironment (TME), resulting in the activation of autologous immune responses (5). Recently, immune checkpoint inhibitors (ICIs), including monoclonal antibodies targeting CTLA-4 and PD-1, have proved to be the greatest breakthrough in the field of tumor therapy. Although collectively ICIs have a response rate of 30–40% (6), a majority of patients lack satisfactory clinical efficacies owing to the complex mechanisms underlying tumor immunity (7). Furthermore, several reported genomic and immune biomarkers indicate that the therapeutic effects are not targeted and there is an inevitable bias whilst evaluating the treatment efficacies (8). Thus, it is challenging but necessary to identify a better predictor to evaluate the clinical outcomes accurately before prescribing ICI treatment.

TME consists of various cell types and an extracellular matrix, thereby supporting tumor behaviors, including their growth and metastases through the provision of energy and nutrients (9). Usually, the blood vessel network in TME is poorly developed or malformed, and thus, exchanging of nutrients and metabolic wastes is relatively impaired. This causes a breakdown of the metabolic balance in the tumor tissues, characterized by nutrition shortage and metabolite accumulation (10). Consequently, the above-mentioned transfer of metabolic mode in TME is a natural immune suppressor, along with the inactivation of immune cells and a decrease in protective inflammatory reactions (11). Additionally, accumulating evidence shows that targeting the metabolic mode in TME is a promising strategy to potentiate the effects of immunotherapy and is therefore worth further investigation.

Excessive production of lactate is the result of elevated aerobic glycolysis in the TME (12). Lactate is responsible for sustaining the acidic environment by decreasing the pH, thereby inhibiting the immune responses partly by inactivating the T cells as also through negative regulation of the T-regulatory cells in the anticancer immunity (13). Meanwhile, a recent study demonstrates that neutralization of the low pH environment in malignant melanoma aids better clinical efficacy of the anti-PD-1 immune strategy (14). In addition, lactate dehydrogenase is being used in clinical settings for the independent prediction of survival of melanoma patients; it is also recommended in the AJCC guidelines (15). Collectively, this indicates that the identification of lactate-related genes (LRGs) for predicting the prognoses in SKCM patients may aid appropriate guidance for therapeutic regimens.

In this study, we analyzed the complete gene expression profiles related to LRGs in 471 patients from The Cancer Genome Atlas (TCGA) database. Six genes were significantly correlated to lactate metabolism as per the Cox regression model. Next, we used the reconstructed model to assess clinical outcomes and responses to immunotherapy among the SKCM patients, and the results showed that this potential strategy may be useful for survival prediction and could be utilized as a novel immune-targeted therapy.

## Materials and Methods

### Data Collection

The transcriptomic profiles of 472 individuals were obtained from TCGA database (https://portal.gdc.cancer.gov), which consisted of data for one healthy skin and 471 SKCM tissues. We then extracted the data for 556 normal skin tissue samples from the Genotype-Tissue Expression Project (GTEx, https://gtexportal.org/home/) web portal to account for the small number of the controls from TCGA database. The gene expression data from TCGA and GTEx were merged and normalized using the “limma” package in R to control for batch effects (16). The abundances of genes were normalized using their fragments per kilobase million (FPKM) values. Furthermore, the GSE54467 (n=79) dataset (17) was extracted from the Gene Expression Omnibus database (GEO, http://www.ncbi.nlm.nih.gov/geo/) and used as an external confirmation cohort to validate the robustness of the gene signature. Patients with entire clinical data as well as those with a survival duration longer than 0 days were included in current research.

### Differential Expression and Functional Enrichment Analyses for LRGs

A total of 184 LRGs were obtained from the Molecular Signatures Database (INCREASED SERUM LACTATE, M35671, http://www.gsea-msigdb.org/gsea/index.jsp) (18). The “limma” package was used to identify the differentially expressed LRGs between SKCM and healthy skin samples with thresholds of |log2 fold change (FC)| ≥ 1 and standard false discovery rate (FDR) < 0.05. The protein-protein interaction (PPI) network of differentially expressed LRGs was predicted using the STRING webtool (https://string-db.org/) (19). The hub sub-modules in the PPI network were selected using the MCODE plug-in in Cytoscape (20). The Gene Ontology (GO) and the Kyoto Encyclopedia of Genes and Genomes (KEGG) enrichment analyses were performed using the “cluster Profiler” package in R (21).

### Consensus Clustering

According to the expression profiles of differentially expressed LRGs in SKCM tissues, consensus clustering was performed using the “ConsensusClusterPlus” package in R by setting the number of groups to 9, the sample resampling to 80%, and the number of iterations to 1000 (22). The optimal cluster number was calculated using the consensus matrix and cumulative distribution function (CDF). The differences in the overall survival (OS) between different clusters were estimated using the Kaplan-Meier method. Comparisons of the distribution of categorical data among the clusters were done using the chi-squared test.

### Construction and Validation of Prognostic LRG Signature

Univariate Cox analysis was employed to identify the differentially expressed LRGs having significant (*P* < 0.05) prognostic prediction value. The selected factors were integrated into the least absolute shrinkage and selection operator (LASSO) Cox regression algorithm and the risk of overfitting was minimized. Lastly, a multivariate Cox regression model was generated for selecting the genes and an LRG-based prognostic model was subsequently established. The risk score for each patient was calculated using the following formula: Risk Score = $\Sigma_{i=1}^{n}Coef(i)\times x(i)$ , where *Coef(i)* and *x(i)* were the regression coefficients in the multivariate Cox regression model and expression of each gene, respectively. The patients were classified either into the high-risk (≥ median number) or the low-risk (< median number) groups according to the median risk score. The survival curve, receiver operating characteristic (ROC) curve, risk score distribution, and heatmap were analyzed and the predictive effectiveness of the clinical signature was thus evaluated. External data from GSE54467 were used to assess the performance of the model in determining clinical outcomes.

For the analysis of the correlation of risk score value based on the signature with clinical parameters in TCGA-SKCM cohort, the chi-square tests were performed. The independence of both the clinical features and the LRG signature was assessed through univariate and multivariate Cox regression analyses. To evaluate the applicability of this signature, stratified Cox survival analysis was performed for subgroups having differential clinical characteristics.

### Development of a Nomogram

Nomograms have been widely adopted as auxiliary tools to predict the individual probability of a clinical event in medical fields (23). Nomogram was built by including all independent prognostic factors (24). In this study, the independent prognostic factors were used to construct the nomogram for assessing the 1-, 3-, and 5-year OS in SKCM. Calibration, ROC, and decision curves were used to verify the ability of the nomogram for predicting the prognoses.

### Functional Biological Analysis of DEGs in the LRG Signature

The differentially expressed genes (DEGs) between the low- and high-risk groups in TCGA-SKCM were analyzed using the “limma” package in R. Genes with |log2FC| ≥ 1 and FDR < 0.05 were identified as significant DEGs and included in the subsequent analysis. GO annotation and KEGG analyses of these DEGs between the two subgroups were performed. Additionally, a gene set enrichment analysis (GSEA) was performed to elucidate the significant functional phenotypes that were significantly different between the risk groups. The GSEA function in Java software was executed and the Hallmark gene set “h.all.v7.4.symbols.gmt” was used (18). The phenotypes with nominal *P* < 0.05 and FDR value < 0.25 were considered statistically significant.

### Immune Infiltration Analysis

To uncover the relationship between the risk score and tumor-infiltrating immune cells, seven algorithms including TIMER (25), CIBERSORT (26), CIBERSORT-ABS, quanTIseq (27), MCP-counter (28), xCELL (29), and EPIC (30) were executed to calculate the immune infiltration values among the samples in TCGA-SKCM cohort. We used a heatmap to show the tumor immune cell infiltration computed using different algorithms for each patient. The Spearman correlation analysis was performed, and the correlation coefficients were presented on a lollipop plot.

Subsequently, single sample GSEA (ssGSEA) was used to quantitate the differences in the infiltration levels of immunocytes between the low- and high-risk subgroups using the “GSVA” package in R (31). The differences among the 16 immune cell types and 13 immune-related pathways were compared between the two subgroups. ESTIMATE was the algorithm that predicted the tumor purity, and the tumor microenvironment scores (including immune score, stromal score, and ESTIMATE score) for each SKCM sample from the gene expression data using the “ESTIMATE” package in R (32). Violin plots were plotted to demonstrate the differences in scores between the two groups.

### Expression of Immune Checkpoint Inhibitors and Immunotherapeutic Responses

To investigate the underlying effects of this signature on the responses to immunotherapy, 47 ICIs were retrieved from published literature, and the expressions of these ICIs between the two groups were analyzed (33). The correlation of the prognostic signature with the expression of two ICIs, including programmed cell death protein 1 (PD-1) and cytotoxic T lymphocyte-associated antigen 4 (CTLA4), was also determined. The immunophenoscore (IPS) algorithms were leveraged to evaluate immunotherapeutic responses as described previously (34).

### Tissue Samples

A total of 15 SKCM tissues and 15 normal skin tissues were obtained from patients received surgery at the Third Affiliated Hospital of Sun Yat-Sen University (Guangzhou, China). None of these patients had received pre-surgery chemotherapy or other treatment. All collected samples were stored in a −80°C refrigerator until further quantitative real-time PCR (qRT-PCR) analysis. The written informed consent was acquired from all subjects, and the present research was approved by the hospital ethical committee.

### Quantitative Real-Time Polymerase Chain Reaction (qRT-PCR)

Total RNA was extracted using the TRIzol reagent (Invitrogen, Grand Island, NY, USA) and reverse transcribed into cDNA using the PrimeScript RT reagent Kit (TaKaRa, Japan) following the manufacturer’s protocols. qRT-PCR was performed with SYBR Green I Master Kit (Roche) on the LightCycler^®^ 480 System (Roche). The relative mRNA levels were normalized against that of GAPDH using the 2^−ΔΔCt^ method. The sequences of the primers used in qRT-PCR are listed in **Table S1**.

### Statistical Analysis

All statistical analyses were performed on R unless indicated otherwise, following the methods described above. *P* < 0.05 was considered statistically significant.

## Results

### Identification of Differentially Expressed LRGs and Functional Enrichment Analysis

**Figure 1:**
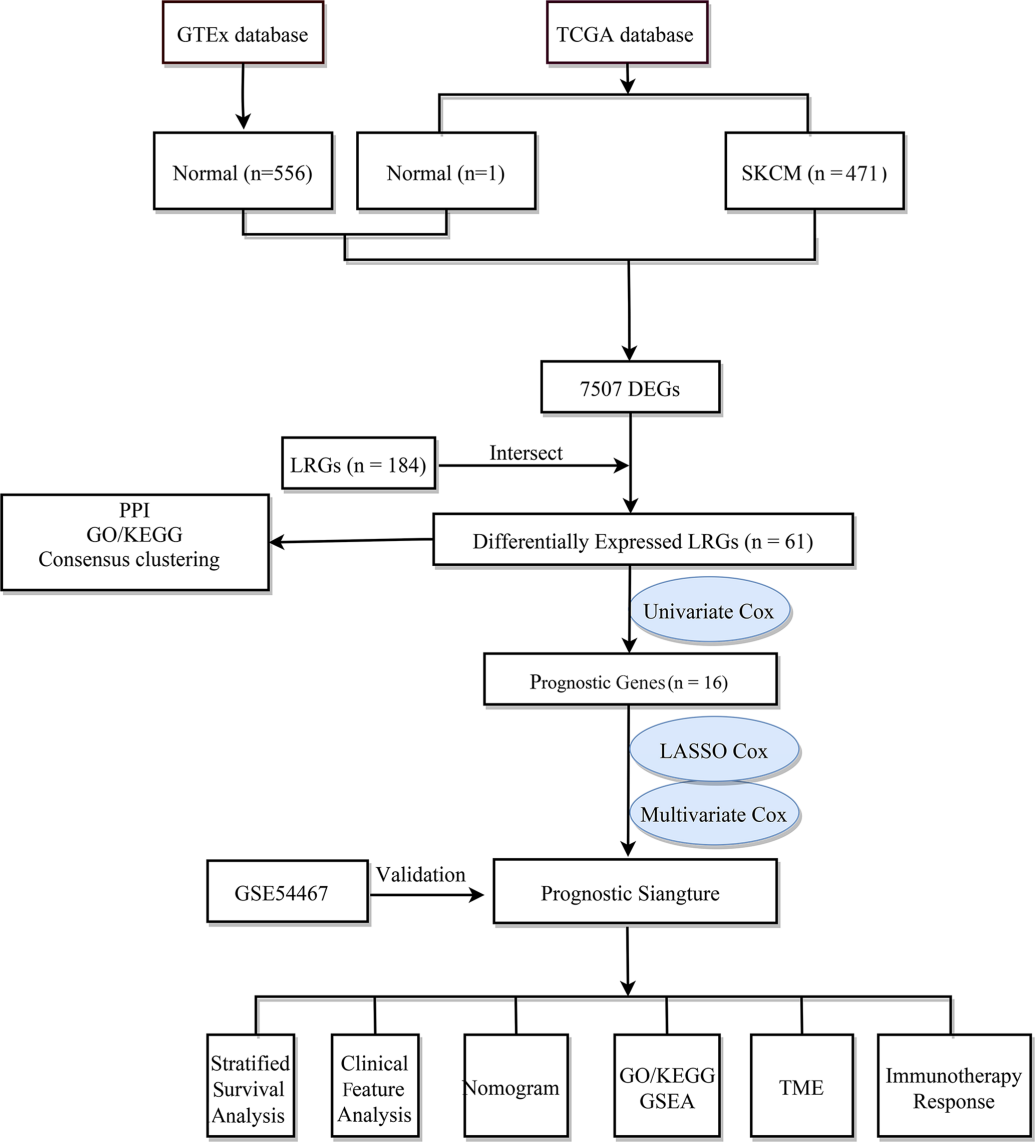
The flow chart of the study design.

**Figure 2:**
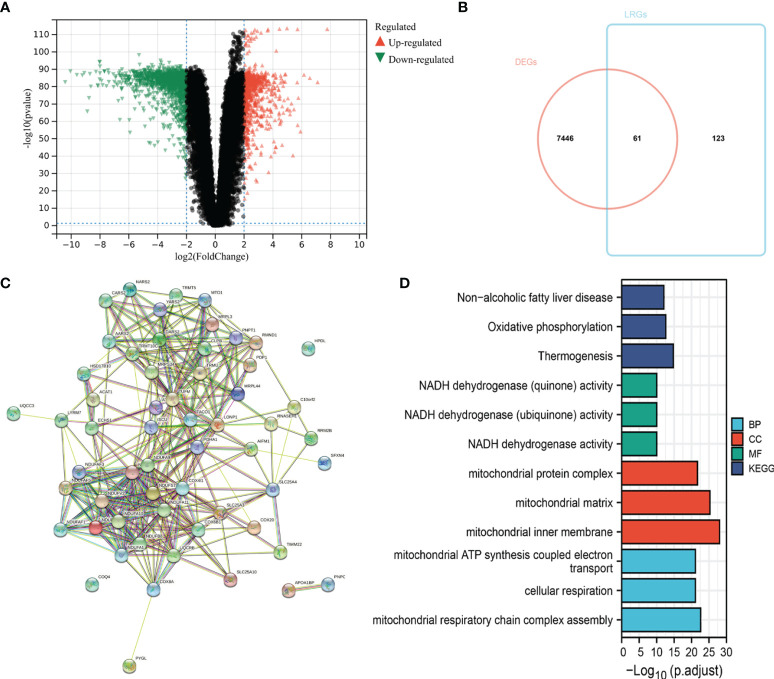
Identification of differentially expressed LRGs in TCGA cohort and functional enrichment analysis. **(A)** Volcano plot showing the DEGs between 471 SKCM and 557 non-tumor healthy samples. **(B)** Venn diagram showing the intersection of DEGs and LRGs. **(C)** The PPI network of differentially expressed LRGs. **(D)** GO and KEGG analyses of differentially expressed LRGs.

The flow chart of the study design is shown in **Figure 1**. First, we analyzed the DEGs between 471 tumor and 557 normal tissues from TCGA and GTEx databases. A total of 7507 DEGs were selected according to the criteria of |log2FC| > 1 and FDR < 0.05. Among them, 3789 DEGs were significantly upregulated in SKCM tissues as compared to the normal skin tissues, while the remaining 3718 were markedly downregulated (**Figure 2A**). In addition, 184 LRGs were obtained from the Molecular Signatures Database. We then acquired 61 differentially expressed LRGs by taking the intersection of DEGs and LRGs sets, which may be involved in the progress of increased serum lactate (**Figure 2B**). A PPI network was constructed for these 61 differentially expressed LRGs (**Figure 2C**). The most significant module was then identified using the MCODE algorithm (**Supplementary Figure 1**). The functions of these 61 differentially expressed LRGs were predicted, and the results of the GO annotation indicated these were markedly augmented in energy metabolism-related processes, including the mitochondrial respiratory chain complex assembly. The differential genes were mostly correlated with pathways of thermogenesis, oxidative phosphorylation, and non-alcoholic fatty liver disease, as evidenced by the KEGG enrichment analysis (**Figure 2D**).

### Determination of SKCM Clusters Using Consensus Clustering

**Figure 3:**
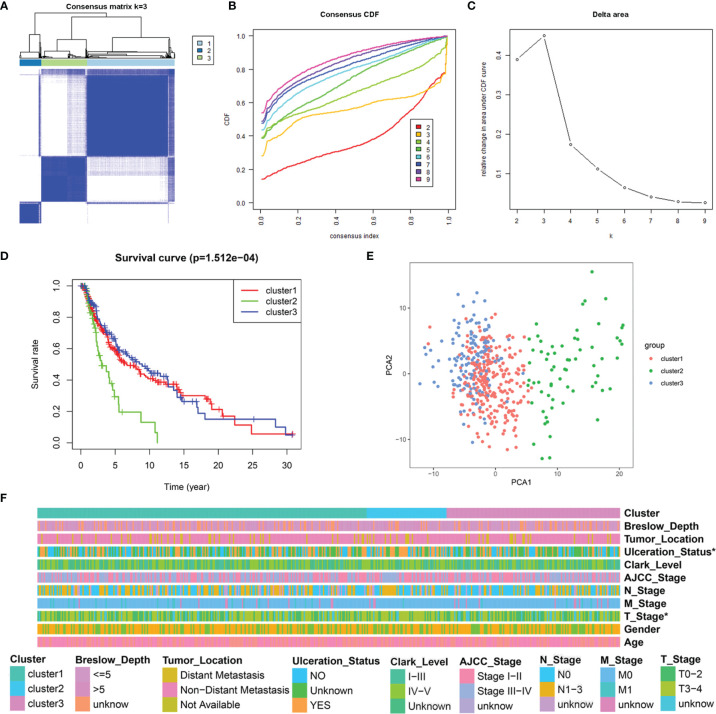
Consensus clustering analysis of 184 LRGs. **(A)** Consensus clustering matrix at K = 3. **(B)** The CDF curves for clusters at k = 2 to 9. **(C)** The relative change in area under CDF curves for different clusters from k = 2 to 9. **(D)** Survival analysis for SKCM samples is stratified to the three clusters. **(E)** PCA plot for the three clusters. **(F)** Heatmap and the clinical parameters of the three clusters. * *P* < 0.05.

To understand the integral role of lactate in SKCM, the SKCM samples were divided into diverse clusters (K = 2 to 9) according to the differential expressions of the 184 LRGs through an unsupervised consensus clustering method. The optimal division (K = 3) was the optimal number of clusters according to the consensus matrix (**Figure 3A**), consensus CDF curves (**Figure 3B**), and relative change in the area under the CDF curves (**Figure 3C**). The boundary of the consensus matrix was kept relatively strict, and the sample distribution reached maximal stability at K =3. A significant difference was observed in the prognoses\ of the SKCM patients, wherein those belonging to cluster 2 suffered poorer outcomes relative to clusters 1 and 3 (**Figure 3D**). In addition, PCA showed that it was feasible to divide the samples into discrete distribution patterns (**Figure 3E**). The chi-square analysis demonstrated statistically significant differences in the T stage (*P* = 0.048) and Ulceration Status (*P* = 0.030) between the SKCM patients and normal controls (**Figure 3F**).

### Construction and Evaluation of the LRG Signature for SKCM

**Figure 4:**
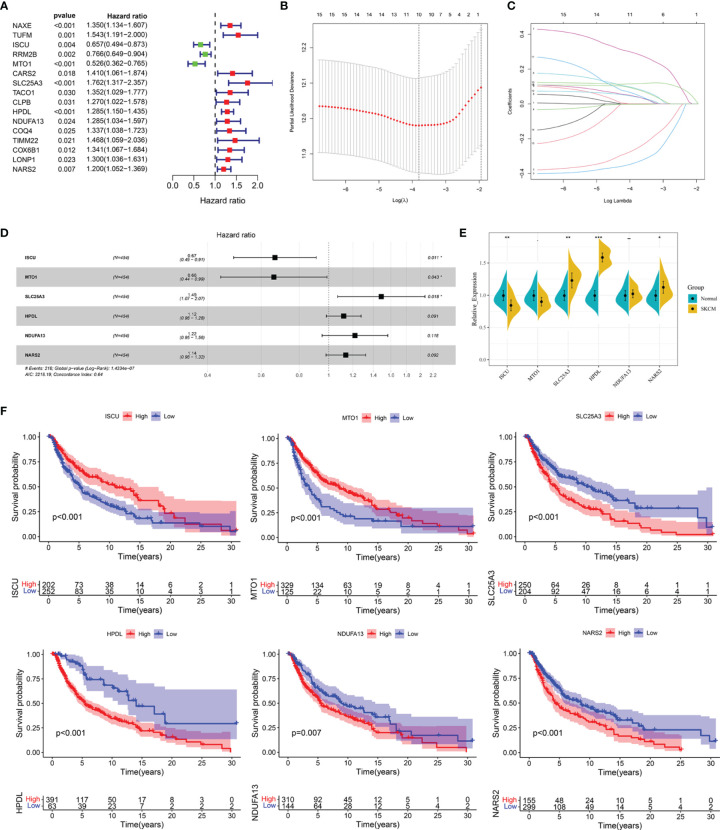
Construction of the LRG prognostic signature in TCGA cohort. **(A)** Identification of the prognosis-related differentially expressed LRGs by univariate Cox regression analysis. **(B, C)** LASSO Cox regression analysis of 16 prognosis-related differentially expressed LRGs. **(D)** Forest plot of the six target genes that compose the LRG signature. **(E)** The expression levels of six target genes by qRT-PCR. **(F)** The Kaplan Meier analysis of the six target genes *P < 0.05; **P < 0.01;***P < 0.001.

Univariate Cox regression analysis showed that 16 out of the 61 differentially expressed LRGs were significantly associated with OS (*P* < 0.05) in TCGA-SKCM cohort (**Figure 4A**). To narrow down the range of candidate genes and eliminate the risk of overfitting, a LASSO Cox regression was performed, and the penalty parameter was selected based on the minimum criterion. A total of 10 genes were retained for further analysis (**Figure 4B, C**) and six target genes (ISCU, MTO1, SLC25A3, HPDL, NDUFA13, and NARS2) were eventually used to construct the LRG prognostic signature based on the multivariate Cox proportional hazards model. The forest map indicated that ISCU and MTO1 were the protective factors with the hazard ratio (HR) < 1, while SLC25A3, HPDL, NDUFA13, and NARS2 were risk factors having a hazard ratio (HR) > 1 (**Figure 4D**). To better understand the role of these six LRGs, we obtained their expressions from the GEPIA database and found markedly low levels of ISCU and MTO1 in SKCM compared with normal samples, while those of SLC25A3, HPDL, NDUFA13, and NARS2 were substantially high (**Supplementary Figure 2**). The results were confirmed by qRT-PCR detection for ISCU, SLC25A3, HPDL, and NARS2, whereas no significant differences were present in the expression of MTO1 and NDUFA13 (**Figure 4E**). The Kaplan Meier survival analysis confirmed the enhanced expression of SLC25A3, HPDL, NDUFA13, and NARS2 which could contribute to the poor outcome of SKCM patients; moreover, high levels of ISCU and MTO1 were significantly associated with better survival in patients (**Figure 4F**), consistent with our previous analysis. For both TCGA and GSE54467 cohorts, the risk score for the LRG signature was calculated as follows: Risk Score = (-0.406 * ISCU_expression_) + (-0.415 * MTO1_expression_) + (0.397 * SLC25A3_expression_) + (0.113 * HPDL_expression_) + (0.198 * NDUFA13_expression_) + (0.129 * NARS2_expression_).

**Figure 5:**
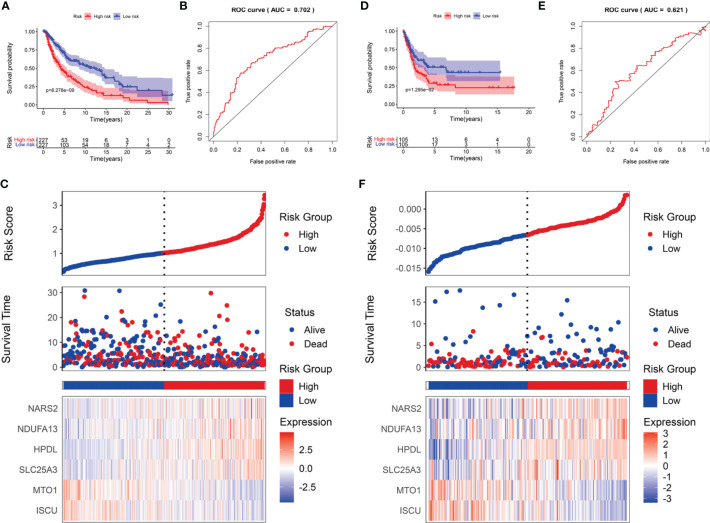
The prognostic value of the LRG signature for SKCM patients. The survival analysis in TCGA cohort **(A)** and GSE54467 cohort **(D)**. ROC curves indicated the predictive efficiency of the prognostic signature in TCGA cohort **(B)** and GSE54467 cohort **(E)**. The risk score distribution, survival status, and heatmap for the expressions of the six genes in TCGA cohort **(C)** and GSE54467 cohort **(F)**.

SKCM cases were divided into low- and high-risk subgroups based on the median risk score. The Kaplan-Meier survival analysis demonstrated that the high-risk subgroup had a shorter OS than that of the low-risk group in TCGA-SCKM (**Figure 5A**) and GSE54467 cohorts (**Figure 5D**). ROC curves were employed to assess the predictive performance of the LRG signature, and the area under the curve (AUC) for TCGA-SKCM was 0.702 (**Figure 5B**). Similarly, the AUC was 0.621 for the GSE54467 cohort (**Figure 5E**). The distribution of the risk score and survival status in TCGA-SKCM are shown in **Figure 5C**. The high-risk group was associated with higher mortality as compared to the low-risk group. Moreover, SLC25A3, HPDL, NDUFA13, and NARS2 were markedly upregulated, while ISCU and MTO1 were substantially downregulated (**Figure 5C**). The results in the GSE54467 cohort were in line with the above-described findings (**Figure 5F**).

### Relationship Between the Risk Score and Clinical Features

**Figure 6:**
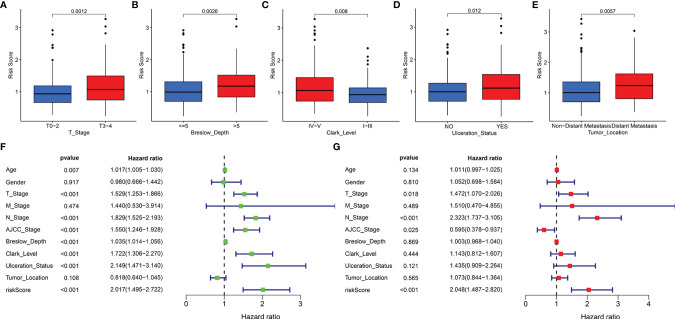
The clinical utility of the LRG signature. The associations between the signature-based risk score and different clinical features, including **(A)** T stage, **(B)** Breslow depth, **(C)** Clark level, **(D)** Ulceration Status, and **(E)** Tumor Location. Univariate **(F)** and multivariate **(G)** Cox analyses for the signature-based risk score and other clinical features in TCGA cohort.

In addition, the correlation of the signature with the clinical features (age, gender, T stage, M stage, N stage, AJCC stage, Breslow depth, Clark level, ulceration status, and tumor location) was tested in TCGA cohort. It was found that the risk scores for the low-risk and the high-risk groups were significantly different for the T stage (**Figure 6A**), Breslow depth (**Figure 6B**), Clark level (**Figure 6C**), ulceration status (**Figure 6D**), and tumor location (**Figure 6E**). We also observed that the SKCM patients in the high-risk group had higher risk factors for disease progression, including advanced T stage, >5mm Breslow depth, IV-V Clark level, with ulceration, and distant metastases. In addition, the signature-based risk score was positively correlated with tumor progression. We then compared the differences in risk scores among the different clusters and found that cluster 2 presented a higher risk score than other clusters, which further verified our results (**Supplementary Figure 3A**). However, there were no significant differences in age, gender, M stage, N stage, and AJCC stage (**Supplementary Figures 3B**–**F**).

We reasonably speculated that the prognostic signature could serve as an independent prognostic factor for patients with SKCM. Therefore, univariate and multivariate Cox regression analyses were performed to confirm this hypothesis. The signature-based risk score was found to be significantly related to OS in univariate Cox analysis (HR = 2.017, *P* < 0.001) (**Figure 6F**). Moreover, multivariate Cox analysis showed that the risk score remained an independent factor (HR = 2.048, *P* < 0.001) (**Figure 6G**). Likewise, the T stage, N stage, and AJCC stage were also independent prognostic factors. Hence, the signature was an independent risk factor that influenced the survival of patients with SKCM.

Further, for validating the stability and applicability of the LRG signature, we performed a stratified survival analysis for the subgroups. In all the subgroups except for the breslow depth > 5 subgroup, the Kaplan-Meier survival curve showed that samples from the high-risk group had poorer clinical outcomes as compared to those belonging to the low-risk group (**Supplementary Figure 4**).

### Construction of the Clinical Nomogram

**Figure 7:**
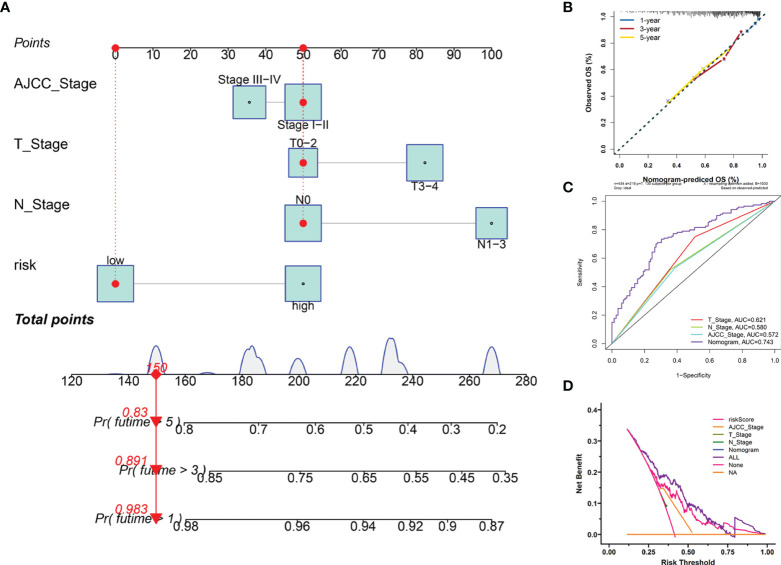
Construction and evaluation of the novel nomogram. **(A)** The nomogram for predicting the survival probability of SKCM patients has four independent prognostic features. **(B)** The calibration plots of the nomogram for predicting OS probability for 1-, 3-, and 5-years. **(C)** ROC analysis of the nomogram. **(D)** DCA of the nomogram.

Furthermore, we employed four independent prognostic features of OS, including the signature-based risk score, T stage, N stage, and AJCC stage to construct the nomogram to quantitatively estimate the 1-, 3-, and 5- year survival probabilities of SKCM patients in TCGA cohort (**Figure 7A**). In the nomogram score system, each variable was allocated a point, and then the sum of the points was calculated as the total score, and the predicted risk corresponding to the total score was the probability of survival (35). The accuracy and sensitivity of the predictions were confirmed using the calibration plot for the nomogram. To intuitively illustrate the performance of the nomogram, calibration curves were plotted which showed that the predicted results were consistent with the reality, thereby suggesting a highly accurate and sensitive prediction for SKCM (**Figure 7B**). The ROC curve analysis showed that the nomogram provided adequate discrimination for the two risk groups with an AUC of 0.743, thereby outperforming other independent clinical prognostic features (T stage, AUC =0.621; N stage, AUC = 0.580; AJCC stage, AUC = 0.572) (**Figure 7C**). The decision curves suggested that the nomogram had the highest overall net benefit within the threshold probabilities relative to any other clinical feature (**Figure 7D**).

### Identification of the Prognostic Signature-Related Biological Processes and Pathways

**Figure 8:**
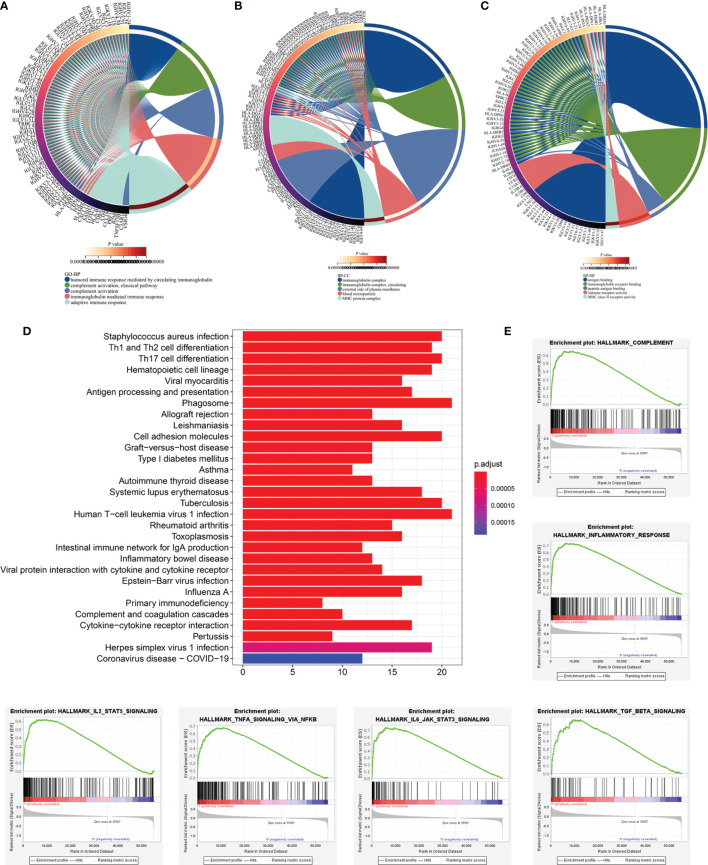
Functional enrichment analyses of DEGs between low‐and high-risk subgroups based on LRG signature. Go annotation terms of DEGs between low‐and high-risk subgroups for biological process **(A)**, cellular components **(B)**, and molecular functions **(C)**. **(D)** KEGG enrichment analysis for DEGs between low‐and high-risk subgroups. **(E)** GSEA findings.

To further detect the biological behaviors that were influenced by the prognostic LRG signature, we identified the DEGs between the low- and high-risk groups to perform the functional enrichment analyses. In total, 252 DEGs were screened for the subsequent analysis based on the criteria of |log2FC| > 1 and FDR < 0.05. The results suggested that the top three enriched GO terms for biological processes (BP) were humoral immune response mediated by circulating immunoglobulin, complement activation-classical pathway, and complement activation (**Figure 8A**). The cellular components (CC) significantly associated with these DEGs included the immunoglobulin complex, immunoglobulin complex-circulation, and lateral side of cytomembrane (**Figure 8B**). The molecular function (MF) analysis showed that the DEGs were related substantially with antigen binding, immunoglobulin receptor binding, and peptide antigen binding (**Figure 8C**). Collectively, the GO annotation suggested that the enrichment of the DEGs was mostly related to the immune-associated processes, which was validated by the KEGG analysis (**Figure 8D**). Besides, we also performed GSEA to compare the different hallmark pathways between the low- and high-risk groups. Most enriched hallmark pathways in the low-risk group were involved in immune regulation, including the complement activation, inflammatory responses, IL2-STAT5 signaling, TNFA signaling *via* NFKB, IL6-JAK-STAT3 signaling, and TGF-beta signaling pathways (**Figure 8E**). These findings suggested that the LRG-based prognostic signature was closely related to immunity and the low‐risk group had enhanced immune response phenotypes.

### Immune Infiltration Characteristics of TME

**Figure 9:**
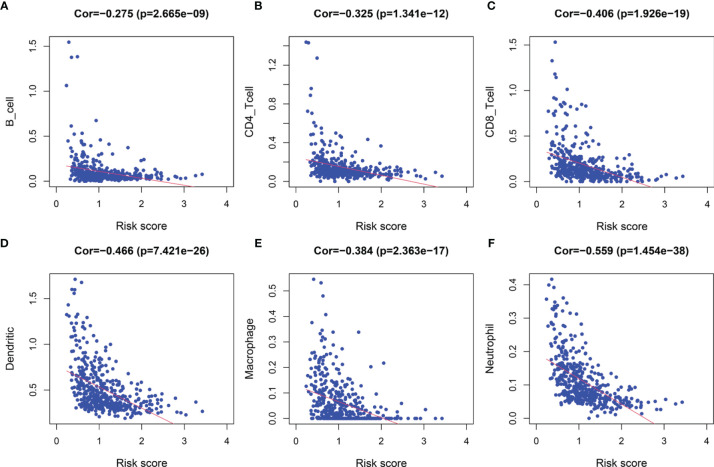
The correlation between the signature and infiltration abundances of six immune cell types. **(A)** B cells, **(B)** CD4+ T cells, **(C)** CD8+ T cells, **(D)** Dendritic cells, **(E)** Macrophages, and **(F)** Neutrophils.

Following the aforementioned results, we postulated that the impact of LRG signature on the outcomes for a patient with SKCM may be associated with the immune microenvironment. Therefore, we assessed the differences in the immune cell components in SKCM tissues between low- and high-risk groups. The heatmap for various immunocyte components based on TIMER, CIBERSORT, CIBERSORT-ABS, quanTIseq, MCP-counter, xCELL and EPIC algorithms, is shown in **Supplementary Figure 5A**. In addition, Spearman correlation analysis was performed, and the correlation coefficients were visualized using a lollipop plot (**Supplementary Figure 5B**). In total, 93 microenvironment components that were examined were found to be diverse between the two groups. Among these, 79 components were negatively correlated with the signature-based risk score, while the remaining 14 were positively correlated. The detailed correlation between the risk score and six immune cell types was computed based on the TIMER database. With an increase in the risk score, there was a marked decrease in the proportion of immunocytes (B cells, CD4+ and CD8+ T cells, dendritic cells, macrophages, and neutrophils) in SKCM patients (**Figures 9A**–**F**).

**Figure 10:**
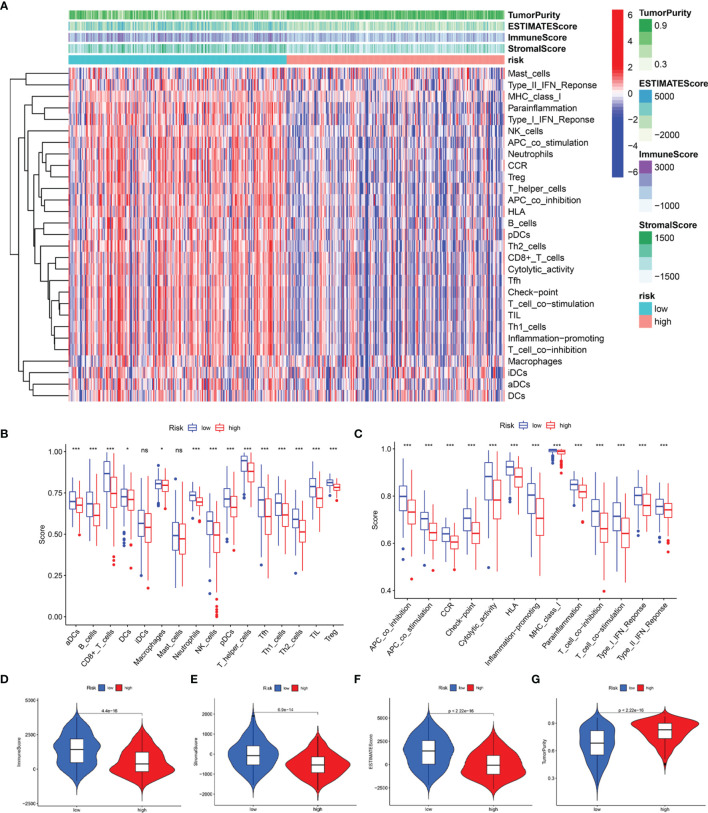
Predicted evaluation of immune microenvironment characteristics. **(A)** Heatmap indicates the scores for tumor purity and the tumor microenvironment between the low- and high-risk groups. **(B)** The differences in the proportions of 16 immune cells between the low- and high-risk groups. **(C)** The differences in the proportions of 13 immune-related pathways between the low- and high-risk groups. The distributions of the immune score **(D)**, stromal score **(E)**, ESTIMATE score **(F)**, and tumor purity **(G)** between the low-and high-risk groups *P < 0.05; ***P < 0.001; ns, no significance.

Subsequently, we estimated the tumor purity and the tumor microenvironment scores using the ESTIMATE algorithm, and the results are shown as a heatmap (**Figure 10A**). The enrichment scores of various immune cell types and immune-related pathways between the two groups were compared. We observed that the abundances of the immune cells except for the iDCs and mast cells (**Figure 10B**), as well as all the immune-related pathways (**Figure 10C**), were markedly elevated in the low-risk group. These results suggested that the two subgroups exhibited distinct immune infiltration profiles. The distributions were then estimated using the ESTIMATE algorithm between the low- and high-risk groups. The immune, stromal, and ESTIMATE scores of the low-risk group were found to be significantly higher relative to the high-risk group (**Figures 10D**–**F**), while the levels of the tumor purity showed a reverse trend (**Figure 10G**). Survival analysis showed that the patients having a higher immune score, higher ESTIMATE score, and lower tumor purity exhibited better prognoses. However, the differences in the stromal scores were not statistically significant (**Supplementary Figure 6**). The above results demonstrated that there was a significant correlation of the signature-based risk score with the tumor immune microenvironment. In addition, the differences in the different immune cell types could account for the observed immune-associated biological phenotypes and pathways related to the LRG signature.

### Differential Expression of ICIs and Assessment of Immunotherapy Response

**Figure 11:**
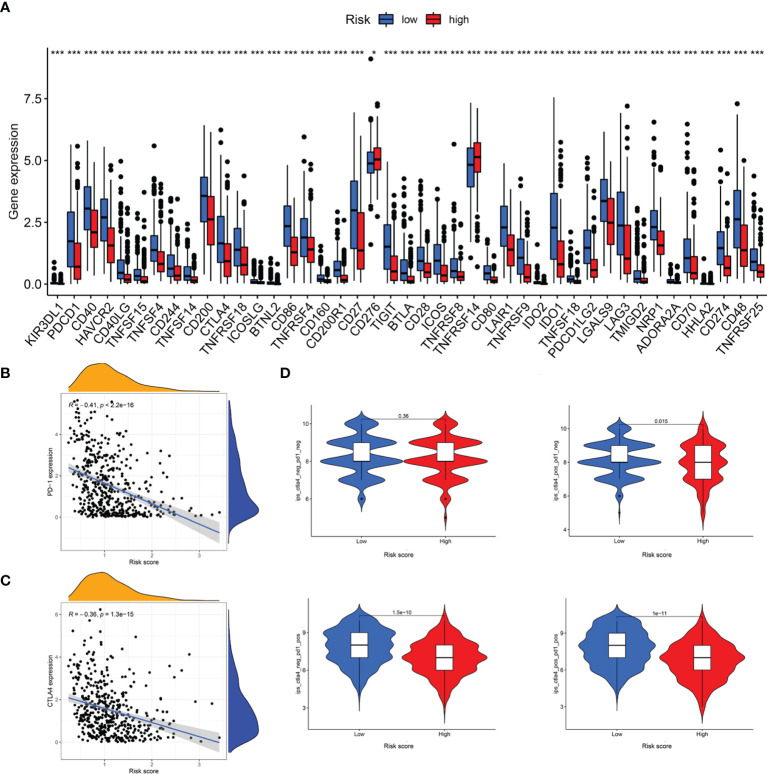
Analysis of immunotherapeutic responses between different risk groups. **(A)** Expression of ICIs in different risk groups. **(B)** The correlation between risk score and PD-1 expression. **(C)** The correlation between risk score and CTLA4 expression. **(D)** IPS scoring scheme estimates the potential responses to immunotherapy in different risk groups *P < 0.05; ***P < 0.001.

The responses to ICI tumor immunotherapy have made important progress in recent years for several cancer types, including SKCM. To further investigate whether the LRG signature was associated with the ICI-related biomarkers, we compared the levels of expression of 47 genes between the two groups and found that 43 ICI-related genes were significantly differentially expressed and all of them were upregulated in the low-risk group relative to the high-risk group, except for CD276 and TNFRSF14 (**Figure 11A**). PD-1 and CTLA-4 are widely studied ICIs. As expected, the levels of expression of these two genes were negatively correlated with the risk score (**Figure 11B, C**). The IPS scoring scheme was used to simulate the potential immunotherapeutic responses in patients of the low- and high-risk groups. The relative probabilities of responding to CTLA4_positive_/PD-1_negtive_, CTLA4_negtive_/PD-1_positive_, and CTLA4_positive_/PD-1_positive_ treatment in the low-risk group were found to be markedly higher relative to the high-risk group (**Figure 11D**). The differences between the two groups for CTLA4_negtive_/PD-1_negtive_ treatment were not statistically significant. Herein, these data demonstrated that the patients with low-risk scores may respond better to the immunotherapy, thereby achieving more satisfactory clinical outcomes.

## Discussion

In our study, we aimed to identify an expression pattern of LRGs, their prognostic value, their impact on the TME, and immunotherapeutic responses in SKCM. First, we identified 61 differentially expressed LRGs by comparing the gene expressions between the SCKM and normal tissues. GO and KEGG enrichment analyses were performed based on these differentially expressed LRGs and the results showed that they were mainly involved in the processes related to energy metabolism. A previous study validates this typical characteristic of tumors, the abnormal energy metabolism, which is substantially different from normal tissues (36). Most tumor cells are highly dependent on aerobic glycolysis, and the remodeling of cellular energy metabolism pathways provides cancer cells with important metabolites, thereby potentiating large-scale biosynthesis, abnormal proliferation, and supporting tumorigenesis. Thus, the inhibition of this metabolic network may serve as a promising therapeutic strategy to selectively kill tumor cells (37).

To further elucidate the relationship between the aforementioned LRGs and survival of patients with SKCM, we determined three subtypes of SKCM, cluster 1, cluster 2, and cluster3, by a consensus clustering method based on the expression profiles of 184 LRGs. The diverse subtypes significantly affected the OS and showed significant differences in clinicopathological features. Specifically, the cases in cluster 2 had poorer prognoses, higher T stage, and with ulceration relative to clusters 1 and 3. Herein, we speculated that lactate metabolism was implicated in the disease progression and clinical outcomes of patients with SKCM.

Next, to evaluate the predictive effect of the LRGs, we constructed a six-LRG prognostic signature by combining Cox regression and Lasso Cox regression analyses. Among the six LRGs, SLC25A3, HPDL, NDUFA13, and NARS2 were risk-associated genes with poorer clinical outcomes, while ISCU and MTO1 were protective factors associated with longer survival duration. Further, we divided the cases into high- and low-risk groups based on the median risk score. As expected, the results of survival analysis were consistent, and the high-risk group presented a significantly worse OS than the low-risk group. Similar results were obtained for the stratified survival analyses among various subgroups. We also observed that SKCM patients belonging to the high-risk group were associated with certain risk factors, for disease progression, including advanced T stage, >5mm Breslow depth, IV-V Clark level, with ulceration, and distant metastases. Univariate and multivariate Cox regression analyses indicated that the signature was an independent risk factor for survival in SKCM.

Some of these six genes comprising the LRG signature have been reported concerning oncogenesis and tumor development. SLC25A3 is a mitochondrial phosphate carrier protein that plays a pivotal role in the aerobic synthesis of the adenosine triphosphate (ATP) (38). Accumulating evidence indicates that homozygous mutations in SLC25A3 are correlated with generalized disorders in mitochondrial-energy metabolism and multisystemic clinical presentation; its high expression is associated negatively with the survival of patients with osteosarcoma (39, 40). 4-hydroxyphenylpyruvate dioxygenase-like protein, HPDL, is a mitochondrial intermembrane space-localized protein that functions as 4-hydroxyphenylpyruvate dioxygenase. It positively regulates mitochondrial bioenergetic processes and ATP generation (41). Meanwhile, HPDL supports tumorigenesis in pancreatic ductal adenocarcinoma in a glutamine metabolism-dependent manner (42). NDUFA13 is a newly identified accessory subunit of mitochondria complex I with a unique molecular structure and a localization that is very close to the subunits of complex I responsible for low electrochemical potential (43). Additionally, it is related to cellular apoptosis in breast cancer (44), the recurrence of prostate cancer (45), and development of squamous cell carcinoma (46). NARS2 is a mitochondrial aminoacyl-tRNA synthetase gene, which encodes a member of the class II family of aminoacyl-tRNA synthetases (47). Mutations in this gene are reported to cause genetic disorders related to neurodegeneration, presenting various clinical features, including refractory seizures, rapid brain atrophy (48), Leigh syndrome, or/and Alpers’ syndrome (49). Its conjoined expression with GAB2 is a risk factor of non- Hodgkin B-cell lymphoma (50). The iron-sulfur cluster assembly protein, ISCU, is engaged in the transportation of iron-sulfur clusters in mitochondrial complex I enzymes, and also functions in mitochondrial respiration for the energy generation (51). Downregulation of ISCU ultimately disrupts mitochondrial energy metabolism, increases the production of mitochondrial reactive oxygen species (ROS), and enhances cell death through the inhibition of complex I. Chen et al. demonstrate that highly upregulated miR-210 can attenuate mitochondrial respiration, thereby resulting in the production of ROS and lactate generation by targeting ISCU, ultimately facilitating the survival of colon cancer cells under a hypoxic microenvironment (52). MTO1 is a mitochondrial tRNA-modifying enzyme that is reported to be a pathogenic factor for mitochondrial disorders (53). However, its expression profile and regulatory mechanisms in cancer have not yet been reported.

Furthermore, we developed the nomogram to quantitatively estimate the 1-, 3-, and 5- year survival probabilities for patients with SKCM by integrating four independent prognostic features, including the risk score. We verified the biological functions related to the prognostic LRG signature through the functional enrichment analysis of 252 DRGs. The results of GO and KEGG enrichment analyses showed that the biological functions were mostly implicated in immune-relevant processes and pathways. Based on the enrichment analysis of the hallmark pathways in diverse risk groups by GSEA, we found that most immune-related signaling pathways were markedly upregulated in the low-risk group, in line with our expectations. Therefore, we speculated that lactate metabolism was closely associated with immune-related processes and signaling pathways, thereby indicating its importance in the progression of SKCM.

As metastatic melanoma is characterized by lymphoid infiltration, it is typically regarded as an immunogenic tumor (54). Therefore, immunotherapy is a prospective therapeutic strategy for metastatic melanoma in addition to surgery, chemotherapy, and target therapy. However, a successful mechanism of action underlying responses to immunological strategy involves several factors, both intrinsic and extrinsic to the cancer cells (55). One of the crucial factors is certainly the TME. Accumulating evidence demonstrates that the biologically significant interaction between tumor tissues and the surrounding microenvironment extensively influences all the phases of the tumorigenic processes (56). Specifically, TME comprises stromal cells, immunocytes, and malignant cells, that collectively interplay with tumor cells and impose many challenges for the initiation, progression, and sensitivity/resistance against the immunotherapy (57). Additionally, a recent study shows that TME supporting tumor growth partly relies on its antitumor immune surveillance and this effect is in part sustained by the abnormal metabolism of tumor cells and cancer-associated fibroblasts in the microenvironment (58, 59).

Given these reasons, the activity of intracellular metabolic pathways of immune cells in TME has drawn widespread attention from researchers. Owing to their special metabolic mode, cancer cells tend to utilize glucose and produce excessive lactate even in an environment with a sufficient oxygen supply and release a large amount of lactate into the extracellular microenvironment, thereby causing acidosis, angiogenesis, and immunosuppression simultaneously (58). Consequently, this kind of metabolism modulation breaks the balance of the immune state in the tumor, resulting in an enhanced immunosuppressive effect by promoting the CD4+ CD25+ regulatory T (Treg) cell metabolic profiles and maintaining the acidity of the TME (60). However, excessive lactate attenuates the proliferation of immunocytes, including CD8+ T, natural killer (NK), and dendritic cells (61–63). Moreover, lactate potentiates the anti-inflammatory effects based on activation of the transformation of macrophages, thereby promoting angiogenesis, tissue remodeling, and finally accelerating tumor growth and invasion (63). Taken together, these results demonstrate that lactate in TME plays a key role in the disease progression and mediating the immunotherapeutic responses.

To date, immunotherapeutic strategies have concentrated on using monoclonal antibodies to activate cell-mediated immunity, also called ICIs (64). Although antibodies against CTLA4 and PD-1, used alone or in combination, both can exert a certain curative effect on the unresectable or metastatic melanoma, the clinical benefits remain unsatisfactory owing to the relatively low ORRs and the phenomenon of drug-resistance (65). Thus, the factors that influence clinical effects and drug resistance of immune strategies should be identified. A previous study demonstrates that the PD-L1 status in the tumor is a biomarker that reflects the response or resistance to ICIs, which was consistent with our conclusion (66). Furthermore, some comprehensive studies have revealed a mechanistically meaningful role of targeting TME, evidenced by the positive association of the ‘T-cell-inflamed tumor microenvironment’ with the effectiveness of diverse immune treatment (67–69).

In our study, we observed that the patients at low-risk tended to present better outcomes and immunotherapeutic responses due to their immune status owing to the TME as compared to the high-risk cases, therefore, in line with the same conclusion as the aforementioned scientific findings. Nevertheless, the main limitation to this study was the lack of experimental data to evaluate the specific mechanism underlying the biological behaviors. Additionally, large-scale multicenter trials are essential to validate the above findings for further clinical application.

In conclusion, we assessed the prognostic significance, effects on the TME, and response to ICIs of LGRs in SKCM. Three subgroups (clusters 1/2/3) identified by consensus clustering based on the expression patterns of LRGs, exhibited dissimilar clinical features. Risk stratification based on the lactate-related prognostic signature was negatively related to clinical prognoses and levels of infiltrating immunocytes in patients. Additionally, the model showed that the low-risk-score patients were likely to benefit more from ICI treatment. Collectively, our findings may be helpful to elucidate the lactate’s role in the TME of SKCM. To sum up, the reconstructed prognostic signature may be applied clinically to survival improvement as well as offer a creative target for curing SKCM patients in the future.

## Data Availability Statement

Publicly available datasets were analyzed in this study. This data can be found here: https://portal.gdc.cancer.gov/, https://gtexportal.org/home/, and https://www.ncbi.nlm.nih.gov/.

## Ethics Statement

The studies involving human participants were reviewed and approved by Third Affiliated Hospital of Sun Yat-Sen University. The patients/participants provided their written informed consent to participate in this study.

## Author Contributions

YX conceived, designed, and wrote the manuscript. JZ and ML assisted in specimen collection and performed experimental work. YZhang and QL were responsible for the data analysis and figures plotted. WL and YZheng helped with manuscript and data review. All authors contributed to the article and approved the submitted version.

## Conflict of Interest

The authors declare that the research was conducted in the absence of any commercial or financial relationships that could be construed as a potential conflict of interest.

## Publisher’s Note

All claims expressed in this article are solely those of the authors and do not necessarily represent those of their affiliated organizations, or those of the publisher, the editors and the reviewers. Any product that may be evaluated in this article, or claim that may be made by its manufacturer, is not guaranteed or endorsed by the publisher.
