# Supplementary material for: Identification of Lactate-Related Gene Signature for Prediction of Progression and Immunotherapeutic Response in Skin Cutaneous Melanoma
Source: Front Oncol. 2022 Feb 21;12:818868. doi: 10.3389/fonc.2022.818868 (PMC8898832; doi:10.3389/fonc.2022.818868)
Supplement: Supplementary file 1 [file DataSheet_1.docx]

**Supplementary materials**

| **Supplementary Table 1. Sequences of primers included in this study.** |
| --- |

| **Genes** | **Forward primer (5’-3’)** | **Reverse primer (5’-3’)** |
| --- | --- | --- |
| SLC25A3 | ATGTCAGCCCTCAACTGGAAG | AATTGGAAATGTACCGCACTCA |
| HPDL | GGGCCTCGAAATGACAGCA | GCCTCCACAATGTTAGGCGTA |
| ISCU | GGGTCCCTTGACAAGACATCT | CCTTTCACCCATTCAGTGGCTA |
| MTO1 | CTCCGCACTTCGACGTGATAG | AATGTCCCTTTCCGATGCCAC |
| NARS2 | TGGGATGGGATTTGAACGCTA | GGAAACCTTGGGAAAGGGATAAC |
| NDUFA13 | GGCCCATCGACTACAAACGG | CGCTCACGGTTCCACTTCATT |
| GAPDH | TGTGGGCATCAATGGATTTGG | ACACCATGTATTCCGGGTCAAT |
|  |  |  |


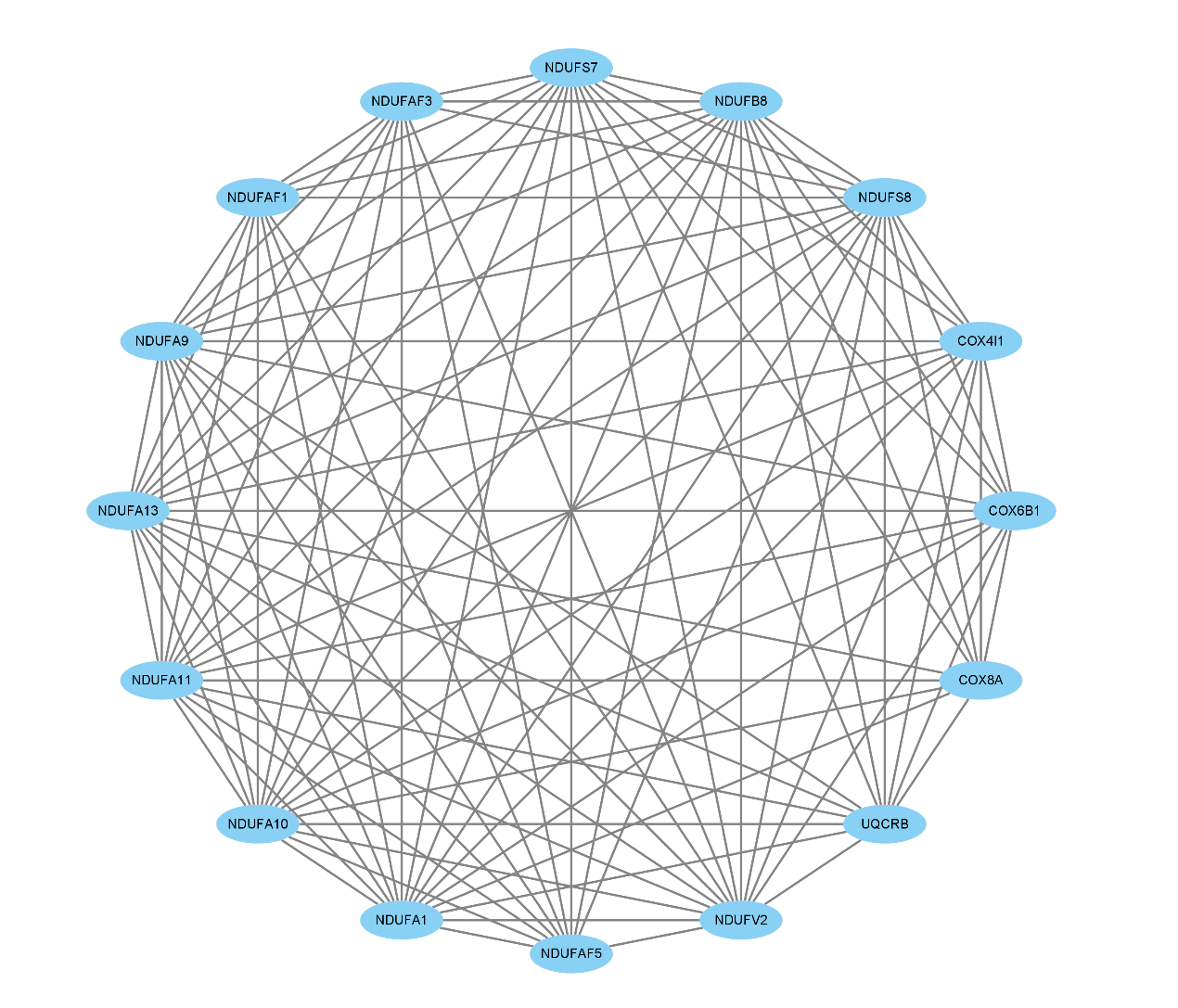


**Supplementary Figure 1. The most significant module of 61 differentially expressed LRGs was then identified using the MCODE algorithm.**


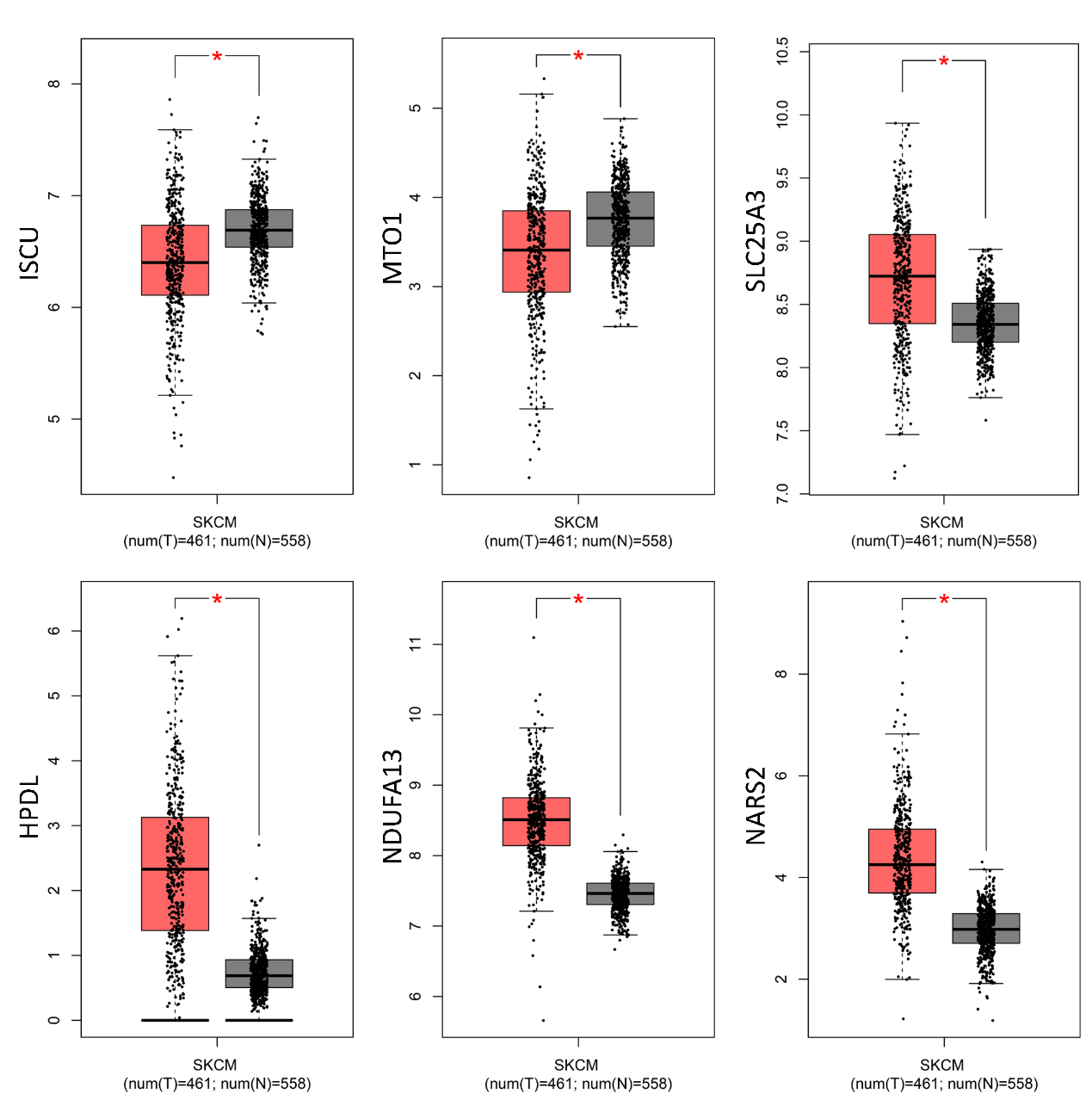


**Supplementary Figure 2. The expression levels of six genes included in the prognostic signature using the GEPIA database.**


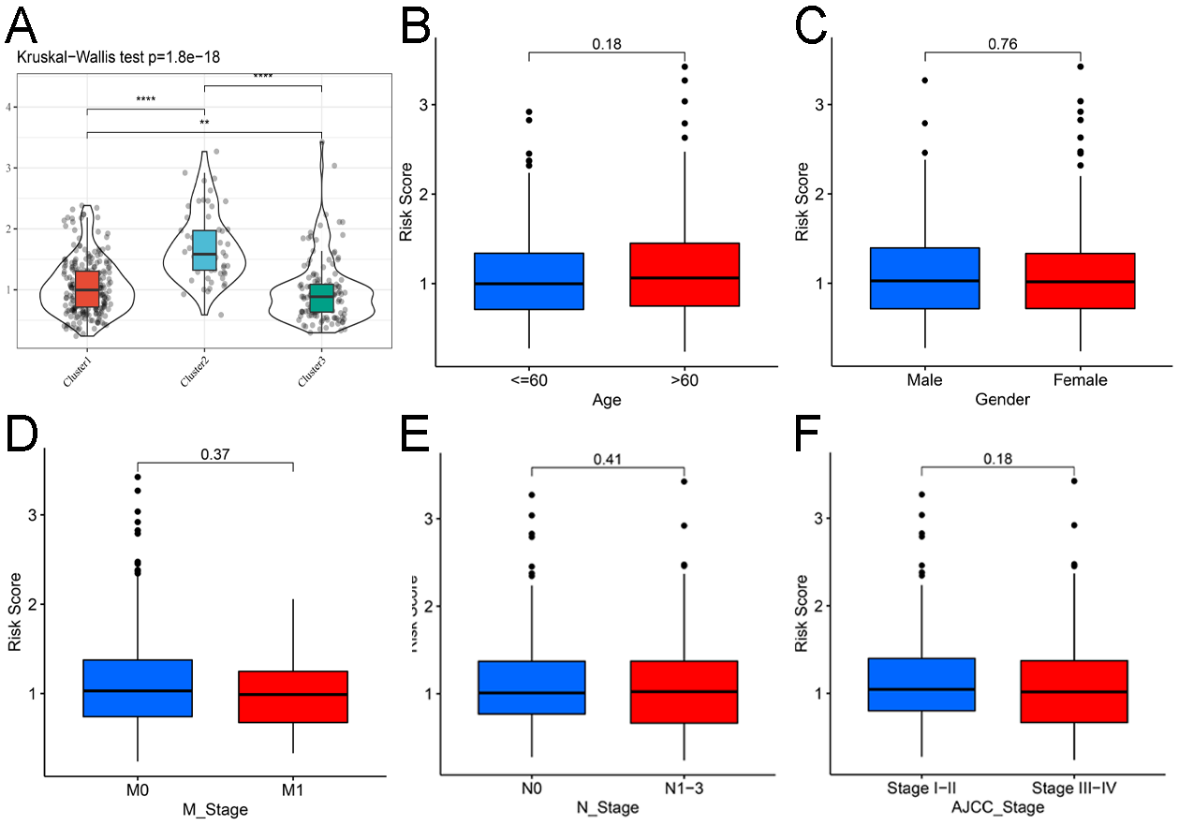


**Supplementary Figure 3. The correlation of clusters and clinical features with LRPS-based risk score.** (A) Clusters, (B) Age, (C) Gender, (D) M stage, (E) N stage, and (F) AJCC stage.


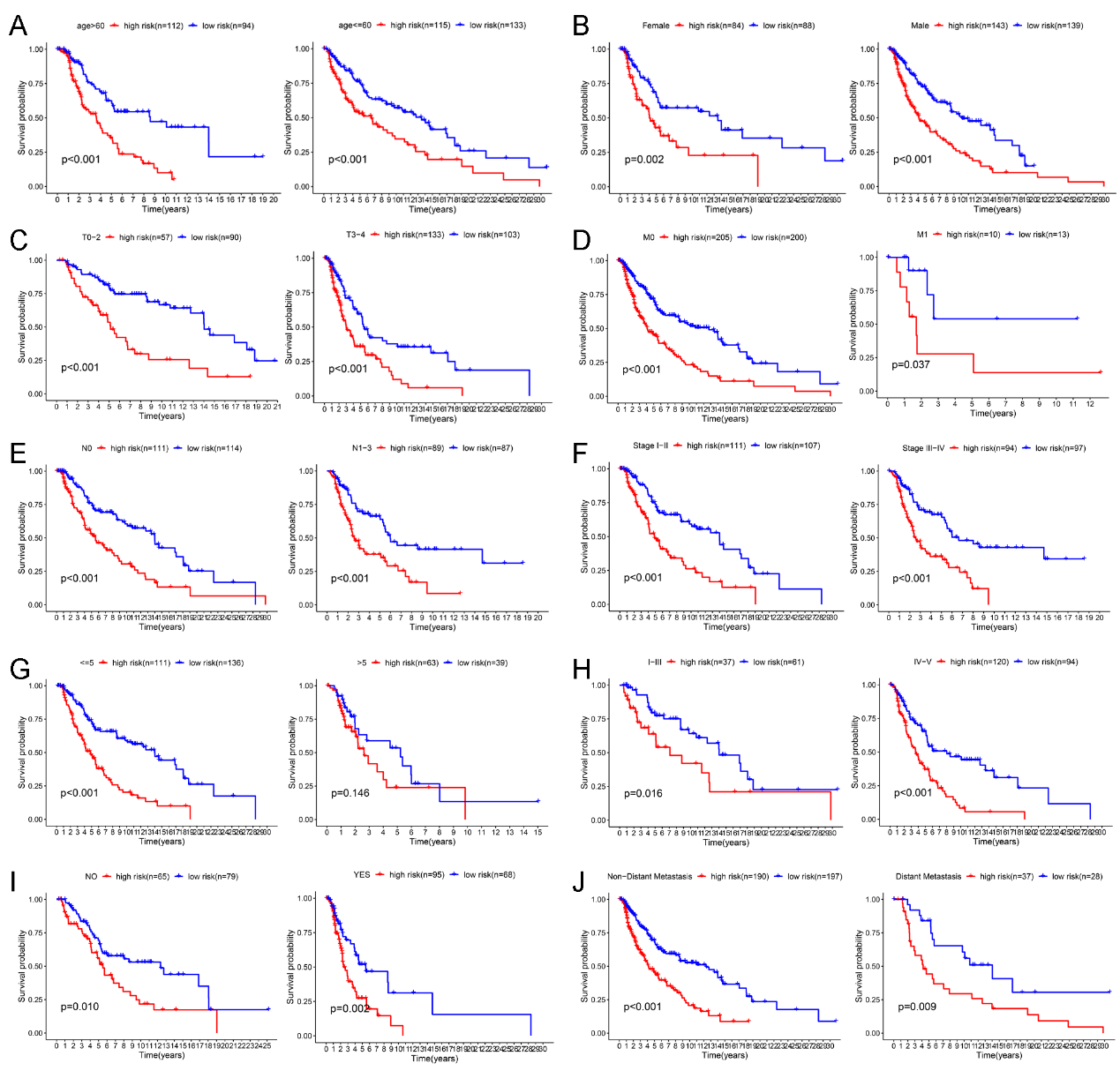


**Supplementary Figure 4. The stratified survival analyses in different subgroups.** (A) age, (B) gender, (C) T stage, (D) M stage, (E) N stage, (F) AJCC stage, (G) Breslow depth, (H) Clark level, (I) ulceration status, and (J) tumor location.


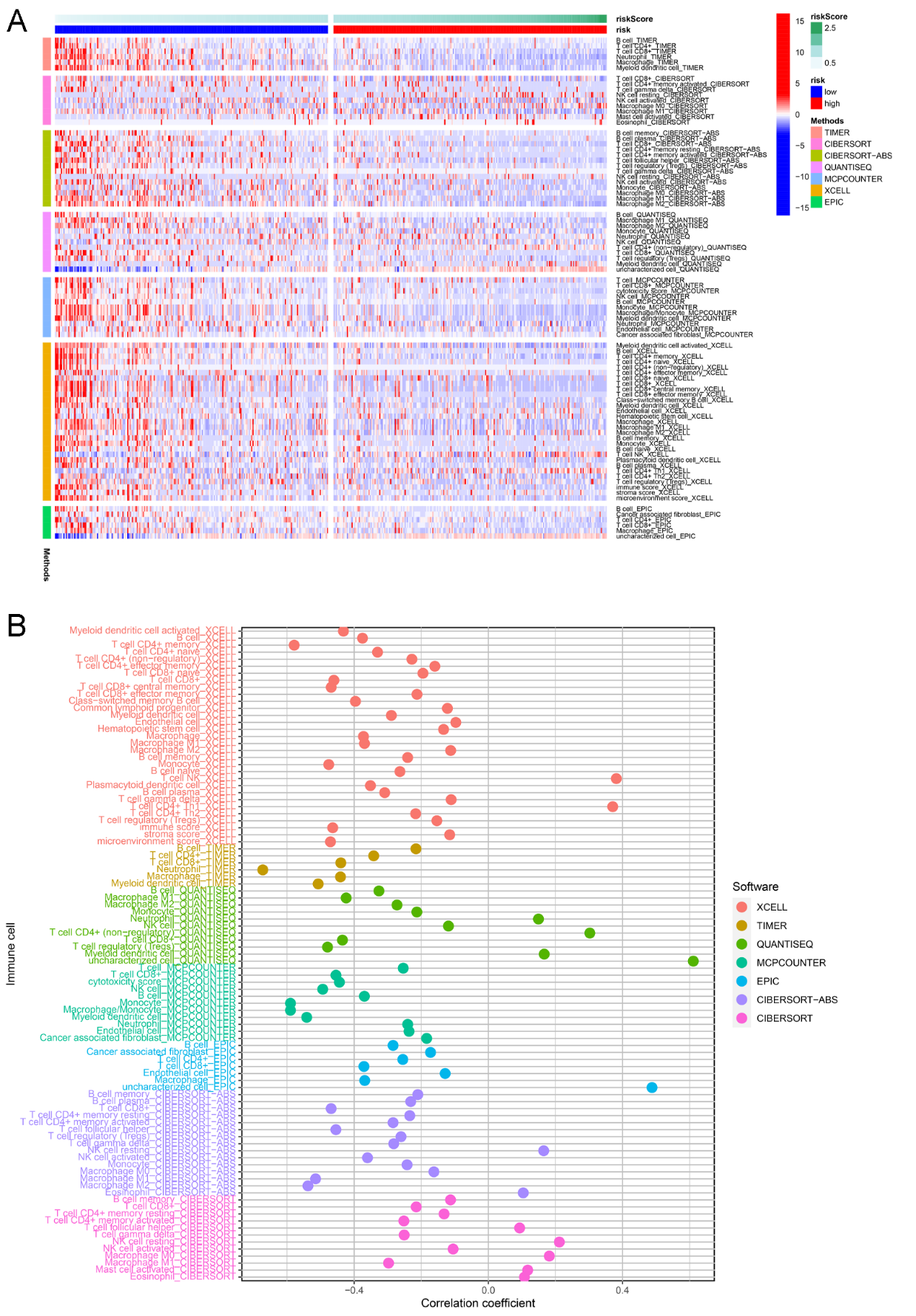


**Supplementary Figure 5. The correlation of tumor-infiltrating cells with LRPS-based risk score using 7 algorithms.** (A) Heatmap. (B) lollipop plot.


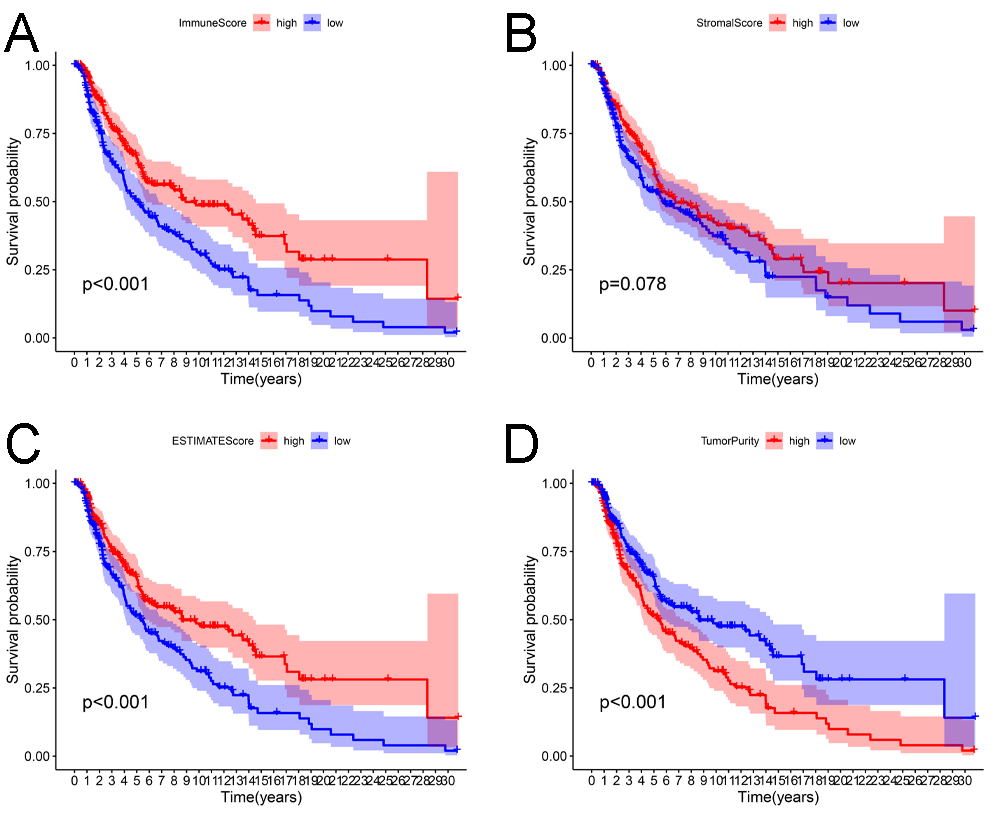


**Supplementary Figure 6. Kaplan-Meier curves of overall survival.** (A) immune score, (B) stromal score, (C) ESTIMATE score, and (D) tumor purity.
